# Geriatric Depression and its Correlates among South Indian Urbans

**DOI:** 10.4172/2167-1044.1000314

**Published:** 2018-08-14

**Authors:** Poojitha Reddy Konda, Pawan Kumar Sharma, Atul R Gandhi, Enakshi Ganguly

**Affiliations:** 1 Mediciti Institute of Medical Sciences, Ghanpur, Hyderabad, India; 2 Department of Epidemiology, University of Pittsburgh, Fogarty International NIH, USA and Share India; 3 Department of Community Medicine, Mediciti Institute of Medical Sciences, Ghanpur, Hyderabad, India; 4 Consultant Statistician & Chief Manager, Monitoring and Evaluation Unit, EdelGive Foundation, Edelweiss House, Mumbai, India

**Keywords:** Geriatric depression, Prevalence, Geriatric depression scale, Urban, Correlates, India

## Abstract

**Background:**

Geriatric depression is a growing global problem, expected to be the leading cause of mortality in the next decade. We attempted to explore the previously unidentified burden of depression and its correlates amongst South Indian elderly residing in an urban area.

**Methods:**

A cross sectional study including 100 community dwelling urban elders aged 60 years and older was conducted. A predesigned questionnaire was used to collect data on sociodemographic variables, chronic health conditions, changes in vision and cognition, addictions, and medication usage. Depression was assessed using Geriatric Depression Scale. Other measurements included anthropometry and blood pressure. Logistic regression was done to identify the independently associated correlates of depression.

**Results:**

The prevalence of geriatric depression was 23%. 15.4% men and 31.2% women had depression. On logistic regression, the independent correlates of depression were living single (OR:4.26; 95% CI:1.06–17.09), poor self-rated health (OR:12.09; 95% CI:1.41–103.14), bedridden (OR:5.29; 95% CI:1.21–23.04) and osteoarthritis (OR: 4.91; 95% CI:1.39–17.28).

**Conclusion:**

The burden of depression in our urban geriatric population was moderate. Several correlates were positively associated. While addressing geriatric morbidity, screening for elderly depression, as well as exploration and management of related factors would be of significance.

## Introduction

Depression is a major public health problem among elderly populations worldwide, with reported global incidences ranging from 1 to 16% [1]. With the projections of increases in global elderly population [2], the common geriatric problems including depression, are expected to compound. In the US, 15–27% of older adults in the community [3] experience depression substantially associated with increases in healthcare costs [4]. The burden is equally high among other populations such as Koreans (15.5%), Japanese (33.5%), Taiwanese (9.8%) and Europeans (12.3%) [5–8]; the European sites showed a wide variation in geriatric depression prevalence with 8.8% in Iceland, 12.0% in Amsterdam, 17.3% in London, and 23.6% in Munich. While the current burden of global depression accounts for approximately 12% of years lived with disability worldwide [9], amounting to approximately US$925 billion, where about 17% of the burden is shared by older adults [10]; it is estimated that the costs may double by 2030 [11]. Moreover, up to two thirds of elderly suicides worldwide are attributed to major depression [12]. Depression is projected to be the second leading cause of world disability by 2020 [13] and another decade thereafter, it is expected to be the largest contributor to disease burden [9]. The problem is expected to be much grave for developing countries including India, the second highest contributor to the world’s geriatric population. The prevalence of elderly depression varies by regions and populations in India, with higher prevalence in urban populations [14]. So far, the few community-based studies from the region have shown the prevalence to be low. One large study from Vellore, India showed a prevalence of any depressive episode (defined using ICD-10 criteria) as 12.7% (95% CI 10.64–14.76%, with mild, moderate and severe depression being 3.2% (95% CI 2.11–4.29%), 7.6% (95% CI 5.96–9.24%) and 1.9% (95% CI 1.05–2.75%) respectively [15]. Another from Mumbai found a prevalence of 45% using the Geriatric Depression Scale [16].

Geriatric depression is both, a disease and risk factor of other diseases. It is a major cause of cognitive dysfunction, dementia, impairing functional activities of daily living and quality of life [17,18]. Depression has been linked to cardiovascular diseases, cognitive decline and multiple morbidities among elderly [19]. In fact, depression has been shown as an intermediary in the pathway of most diseases of geriatric age group, which makes it an important risk factor for all chronic health conditions [20,21]. Several correlations between elderly depression and factors such as low social class, widowed state, unemployment, financial problems, low educational level, living in nuclear family or living alone, presence and number of comorbidities, number of daily medications, urinary incontinence, inadequately fulfilled spiritual needs, patients’ perceived health status and life satisfaction, experiencing hunger, history of cardiac illnesses, transient ischemic attack, past head injury, diabetes and others have been shown earlier [15,20–23]. Physical activity and social support were found to protect against elderly depression [15,20]. The consequences of depression including various health problems, impaired overall functioning and decreased quality of life [24–26] generate the need for its further exploration in different populations.

South Indian population exhibit different characteristics compared to other parts of the country and the world, living in close-knit communities preserving local cultural values around nutrition, mobility and other attributes; and participating actively in social and spiritual life in later years. Based on our previous knowledge of factors associated with depression, we hypothesize that geriatric depression in South Indian elderly may have correlates other than those earlier reported. While depression has been shown to have low prevalence earlier amongst rural elders, matching studies from urban areas are scarce. Moreover, the correlates of depression in this population are not well known. Through the present paper, we aimed to study the prevalence and correlates of geriatric depression among elderly residing in an urban area in South Indians. Our submission to the epidemiology of geriatric depression is expected to help geriatric medicine cater to depressed elderly better and exploration of unidentified depression.

## Research Methodology

### Design and participants

A population-based, analytical, cross-sectional design was followed for this study and it was conducted during later half of 2016 in Hyderabad city of South India, Telangana state. Men and women aged 60 years and older, residing in residential colonies in urban Hyderabad were considered for this study. Ten large residential colonies were randomly selected from 30 listed, from different geographic locations of the city, which adequately represented Hyderabad city. A list of households who had at least one age eligible individual was prepared, and the list was randomised by assigning random number by a random number generator software. Total 124 age eligible individuals were found in 10 residential colonies and 106 were found to be eligible to be enrolled in the study. The response rate was 94.33%. The eligibility criteria were: Men and women aged 60 and more, Indian, apparently healthy, residing in urban residential colonies, possessing ability to understand the investigators’ instructions in English, Hindi or Telugu languages and provided consent to be included in the study. We excluded those who did not give consent, had known neurodegenerative disease, or psychiatric condition, taking psychotropic medication, or living in a nursing home or assisted-care facility. Ethics approval was provided by the Institutional Ethics Committee of MediCiti Institute of Medical Sciences. Written consent from each participant was obtained in participant’s local language.

### Questionnaires and forms

Our forms and questionnaires were designed adapting questionnaires and standard protocols of measurements from large International studies: Health Aging and Body Composition study (Health ABC) and Mobility and Independent Living in Elders Study (MILES) and WHO Study of Global AGEing and Adult Health (SAGE) described elsewhere [27]. Briefly, the questionnaire yielded information on sociodemographic characteristics, self-reported general health, medical history and physical function. The final forms and questionnaires were tested upon 20 peri-urban elderly individuals before use. Construct validity of the tool was ensured by blind back translation into English from local language.

### Measurements

Ascertainment of Geriatric Depression: Depression of participants at households was assessed using Geriatric Depression Scale (GDS) – 15 points [27], the total score ranged from 0 to 15. Higher scores reflected an increase in depressive symptoms and scores ≥ 5 indicated presence of depression; scores 9 to 11 were described as moderate and 12 to 15 was described as severe depression. The Geriatric Depression Scale (GDS) is a valid tool for detecting depression among elderly persons residing in community settings.

Cognitive impairment was assessed by using Mini Mental State Examination (MMSE). This tool used questions and activities to test the orientation, registration, attention and calculation, recall, and language and praxis. A hindi (local Indian language) version of this tool has been tested earlier in the Indian setting and validated. Single cut-off score<24 on MMSE was defined as cognitively impaired for this communication [28].

Quality of sleep was measured by Pittsburgh Sleep Quality Index (PSQI), a self-rated questionnaire which assessed sleep quality and disturbances using nineteen individual items to generate seven “component” scores including subjective sleep quality, sleep latency, sleep duration, habitual sleep efficiency, sleep disturbances, use of sleeping medication, and daytime dysfunction. The sum of scores for these seven components yielded one global score. Participant having scores ≥ 5 were defined as having poor sleep quality [29].

Activities of daily living (ADLs) included difficulty in walking across a small room, bathing, eating, dressing, moving in and out of bed and using toilet, originally described by Katz et al. [30]. Direct questions were asked regarding presence of any difficulty and level of difficulty for all the listed activities. If the participant had difficulty in performing one or more out of these six activities without assistance was considered to be functionally impaired.

### Anthropometry

Height was measured in centimetres using Seca 214 stadiometer (Seca, Hanover, MD). Weight was measured in kilograms using a Seca digital platform scale (Seca 813 Digital scale) with very light clothing. Waist circumference and hip circumference was measured for each participant using a non-flexible fibreglass tape to nearest 1 mm with the respondent standing. In women, the abdominal circumference (waist) was measured as the narrowest part of the body between chest and hips and in men it was measured at the level of the umbilicus. Hip circumference was measured as the maximum circumference around the buttocks posteriorly at the level of greater trochanters (hip bones). A single thin layer of clothing was allowed during hip circumference measurements in view of local dressing norms in order not to violate the dignity of the participants. Waist hip ratio (WHR) and BMI (kg/m2) was calculated. WHR was determined by dividing waist circumference by hip circumference. WHO-recommended WHR cutoff values of 0.90 and 0.80 ≥for men and women respectively were considered for analysis [31]. BMI was defined as the weight in kilograms divided by the square of the height in meters (kg/m2). A BMI of less than 18.5 was classified as underweight, BMI of 18.5 to 24.9 as normal weight, BMI of ≥ 25.0 up to 30.0 was classified as overweight and BMI of ≥ 30.0 was classified as obese [32].

Systolic and diastolic blood pressure was recorded in the left arm in sitting position, using digital monitor, Omron Hem-705 (Omron Healthcare, Inc., Lake Forest, IL). An average of two readings was taken as the final systolic or diastolic blood pressure of the individual.

Other data collected by interview included age, sex, sociodemographic characteristics, years of schooling, occupation, marital status, self-reported general health, addictions, medical history, comorbidities, functional disabilities and medication. The data was collected by a trained investigator who administered the questionnaire to the participant in privacy. Clear instructions were given to the participants at each step for objective measurements as per the standard protocol. If participant failed to hear or understand instructions, the investigator demonstrated the procedure. Each participant completed an interview and measurements in approximately 40 ± 23 minutes.

### Statistical analysis

Data were analysed using Statistical Package for the Social Sciences (SPSS) version 21 software (SPSS Inc., Chicago, IL, USA). We compared characteristics of men vs women and depressed vs non-depressed using chi-square test for categorical variable and t-test for continuous variable and results were reported in proportions and mean and standard deviation. Variables that showed statistical significance (P value<0.10) in the univariate analysis, were considered for multivariate analysis. Precisely we examined age, sex, living single, no education, breathlessness, cognitive impairment, poor self-reported health, poor ADL, bed ridden, osteoarthritis (OA), body aches / pain, calf-pain, not satisfied – current weight and poor sleep variables in multivariate analysis. We excluded variables: difficulty – moving around and knee pain due to high collinearity with ADL and osteoarthritis respectively; and low energy which was part of composite variable of geriatric depression. We did backward elimination logistic regression analysis, the elimination of each variable using a chosen model comparison criterion, eliminating the variable that improved the model the most and repeating this process until no further improvement was possible, to get final set of the independent risk factor variables for depression. Variables with p-value of <0.15 were retained in the final model. We adjusted the multivariate model with age and sex. The results were expressed in Odds ratios (OR); 95% confidence intervals (95% CI).

## Results

The prevalence of geriatric depression at community level was 23%; 15.4% among men and 31.2% among women (p=0.05). The sociodemographic characteristics of the participants stratified by gender are shown in Table 1. We found evidence of differences between men and women in our population for living single (p=0.003), weight (p=0.001), BMI (p<0.001), waist circumference (p<0.001), waist hip ratio (p<0.001), and mean depression scores (p=0.01) (Table 1).

Multiple common geriatric mobility related and medical problems were associated with depression in this population including poor self-reported health (p<0.001), bedridden for past 6 months (p=0.006), arthritis (p=0.005), body aches and pains (p=0.01), knee pain (p=0.03), breathlessness (p=0.03), calf pain (p=0.03), low energy (p<0.001), impaired ADL (p=0.04), not satisfied with body weight (p=0.01), difficulty in moving around (p=0.05), and cognitive impairment (p=0.04) (Table 2).

After controlling for confounders, the overall model of logistic regression showed that depression was significantly independently associated with four correlates: living single (OR:4.26; 95% CI: 1.06–17.09), poor self-rated health (OR:12.09; 95% CI:1.41–103.14), bedridden (OR:5.29; 95% CI:1.21–23.04) and osteoarthritis (OR:4.91; 95% CI:1.39–17.28) (Table 3).

## Discussion

Our study reported depression among elderly to be 23%. Women had nearly double the prevalence (31%) as men (15%) (P=0.05). Our prevalence was higher compared with Netherlands (8.1% having major depression) [33] and China (10.5%) [34]; and lower than South Africa (40%) [35]. Our rates were higher when compared with global median prevalence rate of 10.3% reported by Barua et al. [36] or 1–16% reported by Djernes [1] or independent prospective studies from UK (8.4%) [37] and US (4.2%) [38]. Our rates were very similar to a median prevalence of 21.9% for India, reported by Barua et al., who also found significantly higher proportion of elderly depression in India compared with rest of the world (18.2% vs 5.4%), in a trend analysis between years 1955 to 2005 [36]. Among other Indian studies, two studies similar to ours, one study from Dehradun (Northern India) [39] reported a prevalence of 30%, and another form Bengaluru (Southern Indian) reported 36% [40]; while others from Surat (Central India) [41] and Kolkata (Eastern India) [42] found much higher prevalence of 39% and 50% respectively. Steffens et al. reported similar prevalence of depression (10.19%) among elderly men from the US aged 70 years and more, while the reported prevalence among women was much lower (11.44%) than ours [43]. The methods of screening/ measuring for geriatric depression, as well as the populations however, varied in all the studies with most using the geriatric depression scale while some used other methods. This is of importance since depression, subcortical dementia, and normal aging may all have similar neurobehavioral manifestations, and most cross-sectional studies use instruments that possess limited validity to differentiate between these conditions, thereby inflating rates for depression. Elderly depression in the present study had several independent correlates; living alone (death of spouse), poor self-reported health, bedridden for past 6 months, and having osteoarthritis. One meta-analysis of Indian risk factors for depression reported loss of spouse, living alone, chronic co-morbidities, restricted ADL similar to our study, while the other factors reported by them including older age group, female gender, low socioeconomic status, cognitive impairment were not significant in our study [44]. Independent studies have found associations with education and employment status as well [45], which we did not find.

We found a four folds relationship between depression and living single. Depression among elders living single has been reported earlier by Djernes among elderly from UK, Jonjenelis and colleagues from Netherlands and Padayachey from Durban [1,33,35]. The latter also showed 4-fold increases in risk of depression among widows and widowers. Few Indian studies also reported such associations [41]. Barua et al. also found loss of spouse and living alone to be significantly associated with depression risk [44], while few others reported otherwise, finding no association between the two entities [46]. The World Health Organisation has listed adverse life events including separation, divorce, social isolation, and lack of adequate social support as risk factors for depression among elderly [13]. Depression among elders living alone/single is explainable by their solitude and lack of companionship. This has been shown to reduce social interactions and therefore resulting in depression. Another way of looking at this relationship is the poverty and dependency that follows living single and without social support; poverty has been shown to be a significant risk factor for depression by many [44,47–49].

Poor self-reported health was a significant correlate (0R:12.09, 95% CI:1.41–103.14) of elderly depression in our study. Our findings were confirmed by Huang et al. who in a meta-analysis, showed that elderly with poor self-rated health had 2 folds higher risk for depression compared with those with good self-rated health [50]. Independents studies such as those of Leibson et al. [51] and Mulsant et al. [3] have shown such association earlier. Evidence points to poor self-rated health as a significant predictor of depression among the aged [50]; many however argue for poor self-rated health as a concomitant phenomenon of depression rather than an independent risk factor for increased depression. A study from Durban reported 21 times higher likelihood for depression among elderly with poor health statusSome others, contrary to our finding, did not find these associations to be significant in other populations [52]. Nonetheless, the consistent highly significant association between negative rating of subjective well-being and depression reported by several researchers [35,53] and ours, supports us to propose the determination of subjective health status as a proxy screening measure by peripheral community-based health workers in resource-constrained local settings such as ours, where mental health professionals are scarce; wherein the screen positives may further be referred for formal screening of depression and its management. Such proxy tools have been shown in earlier studies [35] to have better sensitivity (90%) and specificity (~70%) compared to GDS. Being bedridden was related with 5 folds risk of depression in our sample. Of the very few studies reporting such association, Patra et al. showed a direct association of being bedridden with depression among nursing home residents [54]. Cong et al. found a nearly threefold risk (OR: 2.89, 95%CI: 1.03–8.08) of depression among bedridden elderly Chinese [34].

Osteoarthritis, associated with nearly 5 folds risk in our population, has been found to be significantly associated with severe depression by several researchers earlier [55–57]. Depression and chronic pain were shown to be highly prevalent in elderly populations worldwide, with an estimated 13% suffering simultaneously from both conditions [55]. Evidence suggesting neuro inflammation playing a critical role in the pathogenesis of both depression and chronic pain is accumulating, providing sufficient ground for their co-existence, and establishing a bi-directional relationship implying that both may be risk factors for each other. Additionally, both entities have several clinical links, such as gender and site of pain. Body aches and pains have rarely been studied earlier in relation to depression. It is however plausible that, depressed elders frequently complain of multiple vague symptoms, possibly having neuro-biological pathways, for not being actively engaged in personal care and other social activities [58,59]. Since pains cannot be objectively measured, it is difficult to conclude if these pains really lead to depression or vice versa. A relation may however, be drawn with low ADL since the pains can reduce an individual’s capability for self-care. Lenze and colleagues [60] also found a positive association of depression and decreased ADLs earlier, similar to our study.

The above observations and arguments provide substantial ground for proposing future studies of prospective nature to study the inter-relationships between various geriatric attributes and elderly depression. In light of detecting a moderately high prevalence among urban elders in this study, we also propose to routinely screen for elderly depression to initiate timely intervention for its prevention.

### Strengths and Limitations

This is one of the very few studies to report correlates among urban elderly in India. The cross-sectional design of the study limited our capability for temporal associations. The main limitation of the study is its small sample size, which did not allow to compute effect sizes of many of associated parameters, or gender differences reported in global literature. However, during the designing phase, we ensured that the sample was sufficient to detect the prevalence of the key variables of interest. Although we did not initially calculate sample size for detecting depression prevalence and correlates, post hoc power analysis (using SPSS 21.0) however showed that the study had 88.3% power, compared against median global median of 10% reported by Barua et al. [36] to detect prevalence.

## Conclusion

The prevalence of depression in our sample of urban community dwelling elderly was moderately high. Depression was more among women. Living single, poor self-rated health, being bedridden and suffering from osteoarthritis were positively correlated with depression. Owing to the growing burden of geriatric problems, optimum health care of the elderly must focus on depression screen among all elderly, not only for early intervention but also for the understanding of complex neurobehavioral pathways in chronic disease cycles.

## Figures and Tables

**Table 1:** Baseline characteristics of the study population.

Characteristics	Men Percent or Mean ± SD (N =52)	Women Percent or Mean ± SD (N = 48)	Total Percent or Mean ± SD (N=100)	P value
Age (years) (mean)*	70.92 ± 6.76	68.93 ± 7.30	69.97 ± 7.06	0.16
No education (%)	3.8	22.9	13.0	0.005
Marital status (living single) (%)	7.7	31.2	19.0	0.003
Joint family (%)	42.3	39.6	41.0	0.47
Height (cm) (mean)*	164.03 ± 11.00	144.25 ± 15.06	154.58 ± 16.49	<0.001
Weight (kg) (mean)*	70.43 ± 11.88	62.33 ± 12.03	66.50 ± 12.57	0.001
Body Mass Index (kg/m2) (mean)*	26.15 ± 4.10	30.90 ± 8.18	28.45 ± 6.81	<0.001
Waist circumference (cm) (mean)*	93.58 ± 8.54	84.96 ± 11.31	89.40 ± 9.51	<0.001
Hip circumference (cm) (mean)*	101.73 ± 8.30	103.27 ± 10.67	102.47 ± 9.51	0.42
Waist Hip ratio (waist / hip) (mean)*	0.91 ± 0.03	0.82 ± 0.07	0.87 ± 0.07	<0.001
Currently working (%)	3.8	2.1	3.0	0.53
Depression (mean)*	2.96 ± 2.61	4.66 ± 3.95	3.78 ± 3.41	0.01
Depression (GDS > 5) (%)	15.4	31.2	23.0	0.05

X2statistics,

* t test statistics

**Table 2:** Univariate analysis of correlates by status of depression in elders.

Correlates	No Depression Percent or Mean ± SD (n = 77)	Depression Percent or Mean ± SD (n = 23)	P value
Age (years) (mean)*	69.44 ± 7.22	71.73 ± 6.30	0.17
Height (cm) (mean)*	154.71 ± 17.17	154.13 ± 14.35	0.33
Weight (kg) (mean)*	67.24 ± 12.42	64.07 ± 13.04	0.29
Body Mass Index (kg/m^2^) (mean)*	28.82 ± 6.93	27.23 ± 6.41	0.33
Waist circumference (cm) (mean)*	89.84 ± 10.66	87.95 ± 11.51	0.46
Height circumference (cm) (mean)*	102.65 ± 9.55	101.91 ± 9.55	0.48
Marital status (single) (%)	13.0	39.1	0.008
No education (%)	10.4	21.7	0.14
Currently working (%)	96.1	100.0	0.45
Joint family ((%)	41.6	39.1	0.51
Smoking (%)	11.7	4.3	0.27
Alcohol (%)	16.9	8.7	0.27
Self-reported health status (%)	49.4	95.7	<0.001
Bed ridden (past 6 months) (%)	9.1	34.8	0.006
Difficulty in moving around (%)	27.3	47.8	0.05
Difficulty in self-care (%)	6.5	13.0	0.26
Body aches and pains (obstructing daily life work) (%)	20.8	43.5	0.03
Knee pain (significant mobility restriction) (%)	32.5	60.9	0.01
Osteoarthritis (%)	16.9	56.5	<0.001
Stroke (%)	3.9	13.0	0.13
Myocardial Infarction (%)	20.8	21.7	0.56
Angina (%)	22.1	26.1	0.44
Breathlessness (on walking) (%)	10.4	30.4	0.03
Calf / calves pain (%)	18.2	39.1	0.03
Asthma (%)	7.8	8.7	0.59
Hypertension (self-reported + high blood pressure) (%)	68.8	69.9	0.58
Cardiovascular disease (all) (%)	32.5	39.1	0.36
Diabetes (self-reported) (%)	41.6	41.6	0.52
Low energy (self-reported) (%)	19.5	73.9	<0.001
Not satisfied with current weight (%)	11.7	34.8	0.01
Low physical activity (no exercise/no sports) (%)	41.6	56.5	0.15
Systolic Blood pressure (mmHg)(mean)*	134.74 ± 20.33	135.09 ± 16.61	0.94
Diastolic blood pressure (mmHg)(mean)*	78.22 ±10.84	75.67 ± 7.74	0.29
Activities of Daily Living (ADL) (% with difficulty)	29.9	52.2	0.04
Cognitive impairment (%)	6.5	21.7	0.04
Poor Quality of sleep (%)	29.9	47.8	0.09

X2statistics,

* t test statistics

**Table 3:** Logistic regression predicting odds of depression by risk factors in elders.

Risk factors	Odds Ratio (OR)	95% Confidence Interval (CI)	
		Lower	Upper
Osteoarthritis**	4.91	1.39	17.28
Poor health status (self-reported) *	12.09	1.41	103.14
Bed ridden (past 6 months) *	5.29	1.21	23.04
Marital status (living single) *	4.26	1.06	17.09
Not satisfied with current weight*	4.16	0.999	17.35

Variables in the model: (age, sex, living single, no education, breathlessness, cognitive impairment, poor self-reported health, poor ADL, bed ridden, osteoarthritis (OA), body aches/pain, calf-pain, not satisfied – current weight and poor sleep).

* P < 0.05;

** P <0.01
